# (2,4,6-Trimethyl­phen­yl)boronic acid–triphenyl­phosphine oxide (1/1)

**DOI:** 10.1107/S1600536811051609

**Published:** 2011-12-07

**Authors:** Sorin Roşca, Marian Olaru, Ciprian I. Raţ

**Affiliations:** a Universitatea Babeş-Bolyai, Facultatea de Chimie şi Inginerie Chimicã, 11 Arany Janos, 400028 Cluj-Napoca, Romania

## Abstract

In the crystal structure of the title compound, C_9_H_13_BO_2_·C_18_H_15_OP, there are O—H⋯O hydrogen bonds between the O atom of triphenyl­phosphine oxide and one hy­droxy group of the boronic acid. Boronic acid mol­ecules form inversion-related hydrogen-bonded dimers in an *R*_2_^2^(8) motif. The structure is consolidated by inter­molecular C—H⋯O bonds and C—H⋯π inter­actions.

## Related literature

For applications of boronic acids, see: Suzuki (2011 ▶); Yang *et al.* (2011 ▶); Furukawa & Yaghi (2009 ▶). For recently reported structures of triphenyl­phosphine oxide and triphenyl­phos­phine oxide hemihydrate, see: Sivaramkrishna *et al.* (2007 ▶); Ng (2009 ▶). For structures of related boronic acids, see: Filthaus *et al.* (2008 ▶), Cyrański *et al.* (2008 ▶); Rettig & Trotter (1977 ▶).
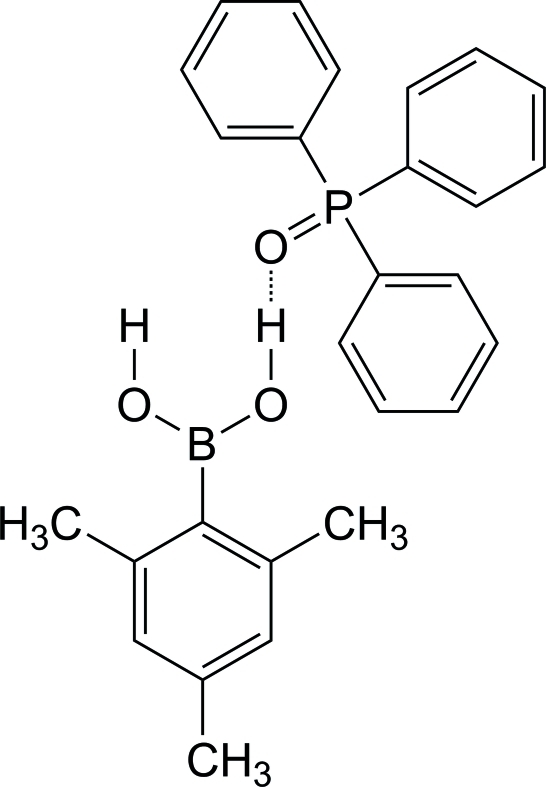


## Experimental

### 

#### Crystal data

C_9_H_13_BO_2_·C_18_H_15_OP
*M* *_r_* = 442.27
Monoclinic, 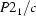

*a* = 12.218 (4) Å
*b* = 12.339 (4) Å
*c* = 16.983 (5) Å
β = 95.651 (5)°
*V* = 2548.0 (14) Å^3^
*Z* = 4
Mo *K*α radiation
μ = 0.13 mm^−1^
*T* = 297 K
0.52 × 0.45 × 0.42 mm

#### Data collection

Bruker SMART CCD area-detector diffractometer
Absorption correction: multi-scan (*SADABS*; Bruker, 2000 ▶) *T*_min_ = 0.934, *T*_max_ = 0.947
23787 measured reflections
4486 independent reflections
3727 reflections with *I* > 2σ(*I*)
*R*_int_ = 0.052

#### Refinement

*R*[*F* ^2^ > 2σ(*F* ^2^)] = 0.083
*wR*(*F* ^2^) = 0.186
*S* = 1.19
4486 reflections
294 parameters
H-atom parameters constrained
Δρ_max_ = 0.40 e Å^−3^
Δρ_min_ = −0.32 e Å^−3^

### 

Data collection: *SMART* (Bruker, 2000 ▶); cell refinement: *SAINT-Plus* (Bruker, 2001 ▶); data reduction: *SAINT-Plus*; program(s) used to solve structure: *SHELXS97* (Sheldrick, 2008 ▶); program(s) used to refine structure: *SHELXL97* (Sheldrick, 2008 ▶); molecular graphics: *DIAMOND* (Brandenburg, 2009 ▶); software used to prepare material for publication: *publCIF* (Westrip, 2010 ▶) and *PLATON* (Spek, 2009 ▶).

## Supplementary Material

Crystal structure: contains datablock(s) I, global. DOI: 10.1107/S1600536811051609/pk2368sup1.cif

Structure factors: contains datablock(s) I. DOI: 10.1107/S1600536811051609/pk2368Isup2.hkl

Supplementary material file. DOI: 10.1107/S1600536811051609/pk2368Isup3.cml

Additional supplementary materials:  crystallographic information; 3D view; checkCIF report

## Figures and Tables

**Table 1:** Hydrogen-bond geometry (Å, °) *Cg*4 is the centroid of the C19–C24 benzene ring.

*D*—H⋯*A*	*D*—H	H⋯*A*	*D*⋯*A*	*D*—H⋯*A*
O2—H2*A*⋯O1	0.82	1.84	2.645 (3)	168
O3—H3*A*⋯O2^i^	0.82	1.99	2.795 (4)	169
C4—H4⋯O1^ii^	0.93	2.41	3.326 (4)	167
C6—H6⋯*Cg*4	0.93	2.88	3.728 (4)	152
C15—H15⋯*Cg*4^iii^	0.93	2.69	3.602 (5)	168

Symmetry codes: (i) 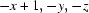; (ii) 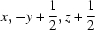; (iii) 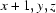.

**Fig. 1.**
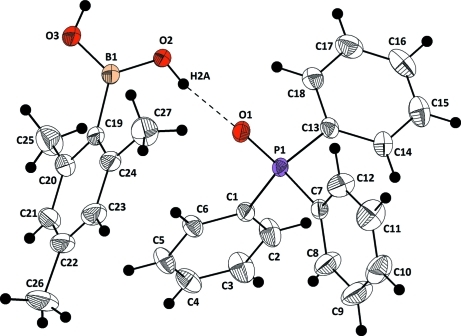
Crystal structure of the title compound with ellipsoids of non-hydrogen atoms drawn at the 25% probability level.

**Fig. 2.**
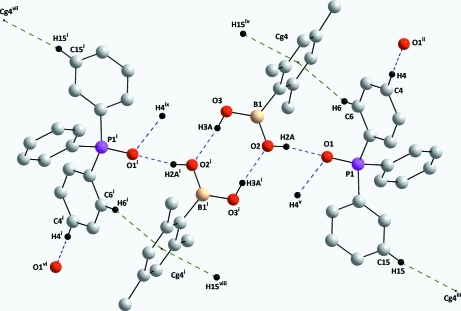
Intramolecular and intermolecular hydrogen bonds and C—H···π interactions in the structure of the title compound shown as dashed lines. Symmetry codes: (i) -x+1, -y, -z; (ii) x, -y+1/2, z+1/2; (iii) x+1, y, z; (iv) x-1, y, z; (v) x, -y+1/2, z-1/2; (vi) -x+1, y-1/2, -z-1/2; (vii) -x, -y, -z; (viii) -x+2, -y, -z; (ix) -x+1, y-1/2, -z+1/2

**Fig. 3.**
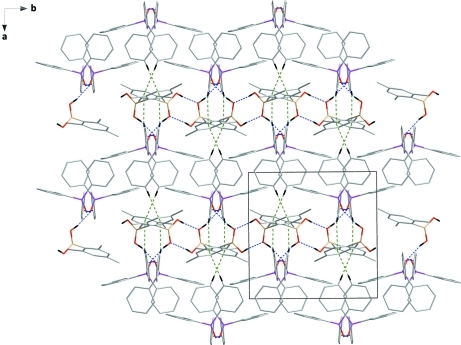
Capped stick representation of the crystal packing of the title compound, viewed along the c axis. Hydrogen bonds and C—H···π interactions shown as dashed lines (blue and green, respectively).

## supplementary crystallographic information

### Comment

Boronic acids are widely used as starting materials in Suzuki-Miyaura cross-coupling reactions (Suzuki, 2011), as sensors or binders for carbohydrates (Yang *et al.*, 2011) or building blocks for covalent organic frameworks (Furukawa & Yaghi, 2009). Although there are a large number of reported crystal structures for aromatic boronic acids, the structure of mesitylboronic acid has not been reported yet.  We present here the crystal structure of the mesityl boronic acid - triphenylphosphine oxide (1:1) adduct.

In the title compound there is an O—H···O bond between the hydrogen atom of a hydroxy group and the oxygen atom of the triphenylphosphine oxide (Fig. 1). The triphenylphosphine oxide molecules are connected into chains by weak C—H···O hydrogen bonds along the *c* axis direction (Table 1 and Fig. 2). The P═O bond length (1.479 (2) Å) is in the range of the values found for triphenylphosphine oxide (1.4863 (12) Å) (Sivaramkrishna *et al.*, 2007), or triphenylphosphine oxide hemidydrate (1.4871 (15) Å) (Ng, 2009).

The molecules of the mesitylboronic acid assemble into centrosymmetric dimers through a pair of O—H···O bonds between the hydroxy groups (Fig. 2). In contrast to the structure of phenylboronic acid where the centrosymmetric dimers are interconnected to one-dimensional chains (Rettig & Trotter, 1977; Cyrański *et al.* 2008), in the structure of the title compound, the triphenylphosphine oxide molecules block further assembly of the dimers.

In comparison to phenylboronic acid, where the angles between the BO_2_ plane and the aromatic ring plane are 6.6° and 21.4° (Rettig & Trotter, 1977) or 6.3° and 21.0° (Cyrański *et al.*, 2008), in the title compound the angle is 75.0 (2)°.  This value is close to the values found for the related pentamethylphenylboronic acid (74.7°, 85.9°) (Filthaus *et al.*, 2008).

The mesitylboronic dimers and the triphenylphosphine oxide chains are interconnected, additionally to the O—H···O bonds, through C—H···π interactions (Table 1 and Fig. 2). In the crystal there are alternate layers of mesitylboronic acid and triphenylphosphine oxide along the *a*-axis (Fig. 3).

### Experimental

The title compound was serendipitously obtained in a Suzuki-Miyaura cross-coupling reaction between mesitylboronic acid and *tert*-butyl *N*-(*tert*-butoxycarbonyl)-*N*-(2,4,6- tribromophenyl)carbamate using tetrakis(triphenlyphosphine)palladium as catalyst. Prior to column chromatography of the crude product mixture a solid precipitated from the mobile phase (diethyl ether: petroleum ether = 1:8).  Colourless crystals were obtained by recrystallization of the precipitate from hot toluene.

### Refinement

Hydrogen atoms were placed in calculated positions with isotropic thermal parameters set at 1.2 times the carbon atoms directly attached for aromatic atoms and 1.5 for hydrogen atoms of the methyl groups and of the hydroxy groups. Methyl hydrogen atoms were allowed to rotate but not to tip. The hydrogen atoms of the hydroxy group were allowed to rotate about the O—B bond and their positions were calculated from the electron density. The C—H bond lengths were set at 0.93 Å for the aromatic groups, 0.96 Å for the methyl groups. The O—H bond lengths were set at 0.82 Å.

### Crystal data

**Table:**

C_9_H_13_BO_2_·C_18_H_15_OP	*F*(000) = 936
*M**_r_* = 442.27	*D*_x_ = 1.153 Mg m^−^^3^
Monoclinic, *P*2_1_/*c*	Melting point: 401 K
Hall symbol: -P 2ybc	Mo *K*α radiation, λ = 0.71073 Å
*a* = 12.218 (4) Å	Cell parameters from 7170 reflections
*b* = 12.339 (4) Å	θ = 2.2–26.0°
*c* = 16.983 (5) Å	µ = 0.13 mm^−^^1^
β = 95.651 (5)°	*T* = 297 K
*V* = 2548.0 (14) Å^3^	Block, colourless
*Z* = 4	0.52 × 0.45 × 0.42 mm

### Data collection

**Table:**

Bruker SMART CCD area-detector diffractometer	4486 independent reflections
Radiation source: fine-focus sealed tube	3727 reflections with *I* > 2σ(*I*)
graphite	*R*_int_ = 0.052
φ and ω scans	θ_max_ = 25.0°, θ_min_ = 1.7°
Absorption correction: multi-scan (*SADABS*; Bruker, 2000)	*h* = −14→14
*T*_min_ = 0.934, *T*_max_ = 0.947	*k* = −14→14
23787 measured reflections	*l* = −20→20

### Refinement

**Table:**

Refinement on *F*^2^	Primary atom site location: structure-invariant direct methods
Least-squares matrix: full	Secondary atom site location: difference Fourier map
*R*[*F*^2^ > 2σ(*F*^2^)] = 0.083	Hydrogen site location: inferred from neighbouring sites
*wR*(*F*^2^) = 0.186	H-atom parameters constrained
*S* = 1.19	*w* = 1/[σ^2^(*F*_o_^2^) + (0.0654*P*)^2^ + 1.8657*P*] where *P* = (*F*_o_^2^ + 2*F*_c_^2^)/3
4486 reflections	(Δ/σ)_max_ < 0.001
294 parameters	Δρ_max_ = 0.40 e Å^−^^3^
0 restraints	Δρ_min_ = −0.32 e Å^−^^3^

### Special details

**Table:**

Geometry. All e.s.d.'s (except the e.s.d. in the dihedral angle between two l.s. planes) are estimated using the full covariance matrix. The cell e.s.d.'s are taken into account individually in the estimation of e.s.d.'s in distances, angles and torsion angles; correlations between e.s.d.'s in cell parameters are only used when they are defined by crystal symmetry. An approximate (isotropic) treatment of cell e.s.d.'s is used for estimating e.s.d.'s involving l.s. planes.
Refinement. Refinement of *F*^2^ against ALL reflections. The weighted *R*-factor *wR* and goodness of fit *S* are based on *F*^2^, conventional *R*-factors *R* are based on *F*, with *F* set to zero for negative *F*^2^. The threshold expression of *F*^2^ > σ(*F*^2^) is used only for calculating *R*-factors(gt) *etc*. and is not relevant to the choice of reflections for refinement. *R*-factors based on *F*^2^ are statistically about twice as large as those based on *F*, and *R*- factors based on ALL data will be even larger.

### Fractional atomic coordinates and isotropic or equivalent isotropic displacement parameters (Å^2^)

**Table:**

	*x*	*y*	*z*	*U*_iso_*/*U*_eq_	
B1	0.4462 (3)	0.1262 (3)	0.0679 (3)	0.0532 (10)	
C1	0.7708 (3)	0.2938 (3)	0.24399 (18)	0.0454 (8)	
C2	0.8540 (3)	0.2801 (4)	0.3042 (2)	0.0760 (13)	
H2	0.9264	0.2729	0.2923	0.091*	
C3	0.8303 (4)	0.2772 (4)	0.3817 (2)	0.0881 (15)	
H3	0.887	0.2698	0.4221	0.106*	
C4	0.7244 (4)	0.2850 (4)	0.3997 (2)	0.0732 (12)	
H4	0.7085	0.2809	0.4521	0.088*	
C5	0.6422 (3)	0.2990 (4)	0.3407 (2)	0.0711 (12)	
H5	0.57	0.3057	0.353	0.085*	
C6	0.6645 (3)	0.3033 (3)	0.2629 (2)	0.0558 (9)	
H6	0.6074	0.3127	0.2231	0.067*	
C7	0.8321 (3)	0.4379 (3)	0.12130 (19)	0.0445 (8)	
C8	0.8551 (4)	0.5149 (4)	0.1778 (3)	0.0886 (15)	
H8	0.8539	0.4969	0.2309	0.106*	
C9	0.8799 (5)	0.6187 (5)	0.1572 (3)	0.116 (2)	
H9	0.8944	0.6704	0.1966	0.14*	
C10	0.8837 (4)	0.6475 (4)	0.0815 (4)	0.0943 (16)	
H10	0.9024	0.7178	0.0684	0.113*	
C11	0.8599 (4)	0.5720 (4)	0.0243 (3)	0.0932 (15)	
H11	0.8619	0.5909	−0.0285	0.112*	
C12	0.8328 (4)	0.4686 (3)	0.0436 (2)	0.0732 (12)	
H12	0.8147	0.4184	0.0036	0.088*	
C13	0.9137 (3)	0.2202 (3)	0.12764 (18)	0.0462 (8)	
C14	1.0189 (3)	0.2615 (3)	0.1331 (2)	0.0600 (10)	
H14	1.0307	0.3341	0.1458	0.072*	
C15	1.1067 (3)	0.1965 (5)	0.1199 (3)	0.0791 (13)	
H15	1.1773	0.2254	0.1232	0.095*	
C16	1.0903 (4)	0.0898 (5)	0.1019 (3)	0.0851 (14)	
H16	1.1499	0.0462	0.0929	0.102*	
C17	0.9869 (4)	0.0465 (4)	0.0970 (3)	0.0833 (14)	
H17	0.9762	−0.0267	0.0856	0.1*	
C18	0.8983 (3)	0.1118 (3)	0.1091 (2)	0.0654 (11)	
H18	0.8278	0.0826	0.1048	0.078*	
C19	0.4131 (2)	0.2237 (3)	0.1205 (2)	0.0499 (9)	
C20	0.3890 (3)	0.2063 (3)	0.1978 (3)	0.0649 (11)	
C21	0.3536 (3)	0.2925 (4)	0.2418 (3)	0.0792 (14)	
H21	0.3389	0.2805	0.2938	0.095*	
C22	0.3398 (4)	0.3942 (4)	0.2105 (4)	0.0868 (16)	
C23	0.3665 (3)	0.4111 (4)	0.1346 (3)	0.0799 (14)	
H23	0.3603	0.4805	0.1133	0.096*	
C24	0.4023 (3)	0.3276 (3)	0.0892 (3)	0.0613 (10)	
C25	0.4022 (4)	0.0969 (5)	0.2351 (3)	0.1026 (17)	
H25A	0.4762	0.072	0.2326	0.154*	
H25B	0.3519	0.0471	0.2074	0.154*	
H25C	0.387	0.1013	0.2894	0.154*	
C26	0.2944 (5)	0.4862 (5)	0.2583 (4)	0.139 (3)	
H26A	0.3226	0.4794	0.3129	0.209*	
H26B	0.2156	0.4823	0.2538	0.209*	
H26C	0.3167	0.5546	0.2382	0.209*	
C27	0.4303 (4)	0.3506 (4)	0.0065 (3)	0.0922 (15)	
H27A	0.5088	0.3519	0.0059	0.138*	
H27B	0.4003	0.4195	−0.0105	0.138*	
H27C	0.3997	0.2949	−0.0285	0.138*	
O1	0.69683 (17)	0.2649 (2)	0.08921 (13)	0.0520 (6)	
O2	0.55104 (18)	0.1109 (2)	0.04987 (17)	0.0615 (7)	
H2A	0.5885	0.1638	0.0642	0.092*	
O3	0.3685 (2)	0.0561 (2)	0.0395 (2)	0.0792 (9)	
H3A	0.3946	0.0134	0.0095	0.119*	
P1	0.79424 (7)	0.30091 (7)	0.14123 (5)	0.0403 (3)	

### Atomic displacement parameters (Å^2^)

**Table:**

	*U*^11^	*U*^22^	*U*^33^	*U*^12^	*U*^13^	*U*^23^
B1	0.042 (2)	0.049 (2)	0.071 (3)	−0.0051 (18)	0.0146 (19)	−0.011 (2)
C1	0.0455 (19)	0.054 (2)	0.0379 (17)	−0.0065 (16)	0.0080 (14)	−0.0018 (15)
C2	0.054 (2)	0.133 (4)	0.041 (2)	0.003 (2)	0.0049 (17)	0.008 (2)
C3	0.077 (3)	0.147 (5)	0.040 (2)	0.011 (3)	0.002 (2)	0.015 (3)
C4	0.092 (3)	0.089 (3)	0.043 (2)	0.009 (3)	0.027 (2)	0.006 (2)
C5	0.068 (3)	0.096 (3)	0.054 (2)	0.008 (2)	0.028 (2)	0.008 (2)
C6	0.056 (2)	0.067 (2)	0.046 (2)	0.0010 (18)	0.0137 (16)	0.0003 (18)
C7	0.0386 (17)	0.054 (2)	0.0414 (18)	−0.0058 (15)	0.0071 (14)	−0.0007 (16)
C8	0.121 (4)	0.086 (3)	0.060 (3)	−0.052 (3)	0.016 (3)	−0.010 (2)
C9	0.169 (6)	0.092 (4)	0.091 (4)	−0.069 (4)	0.028 (4)	−0.024 (3)
C10	0.100 (4)	0.060 (3)	0.123 (5)	−0.026 (3)	0.013 (3)	0.012 (3)
C11	0.112 (4)	0.088 (4)	0.079 (3)	−0.014 (3)	0.002 (3)	0.031 (3)
C12	0.101 (3)	0.063 (3)	0.054 (2)	−0.013 (2)	0.001 (2)	0.009 (2)
C13	0.049 (2)	0.056 (2)	0.0354 (17)	−0.0041 (16)	0.0121 (14)	0.0053 (15)
C14	0.045 (2)	0.070 (3)	0.066 (2)	−0.0018 (18)	0.0091 (17)	0.008 (2)
C15	0.053 (2)	0.109 (4)	0.076 (3)	0.010 (3)	0.011 (2)	0.021 (3)
C16	0.083 (3)	0.106 (4)	0.069 (3)	0.039 (3)	0.024 (2)	0.011 (3)
C17	0.101 (4)	0.064 (3)	0.088 (3)	0.012 (3)	0.026 (3)	−0.006 (2)
C18	0.068 (3)	0.063 (3)	0.068 (3)	−0.006 (2)	0.022 (2)	−0.004 (2)
C19	0.0327 (17)	0.047 (2)	0.071 (2)	−0.0016 (14)	0.0082 (16)	−0.0158 (18)
C20	0.047 (2)	0.071 (3)	0.078 (3)	0.0016 (19)	0.0128 (19)	−0.014 (2)
C21	0.056 (2)	0.106 (4)	0.078 (3)	0.000 (2)	0.014 (2)	−0.037 (3)
C22	0.061 (3)	0.079 (4)	0.121 (4)	0.003 (2)	0.010 (3)	−0.051 (3)
C23	0.069 (3)	0.051 (2)	0.119 (4)	0.005 (2)	0.006 (3)	−0.023 (3)
C24	0.049 (2)	0.052 (2)	0.083 (3)	0.0019 (17)	0.0062 (19)	−0.016 (2)
C25	0.106 (4)	0.106 (4)	0.101 (4)	0.004 (3)	0.037 (3)	0.007 (3)
C26	0.121 (5)	0.121 (5)	0.180 (7)	0.017 (4)	0.034 (4)	−0.094 (5)
C27	0.092 (3)	0.078 (3)	0.108 (4)	0.008 (3)	0.019 (3)	0.013 (3)
O1	0.0442 (13)	0.0712 (16)	0.0411 (13)	−0.0167 (11)	0.0067 (10)	−0.0048 (11)
O2	0.0423 (14)	0.0562 (16)	0.0888 (19)	−0.0107 (11)	0.0206 (13)	−0.0316 (14)
O3	0.0438 (14)	0.0710 (19)	0.126 (3)	−0.0144 (13)	0.0257 (15)	−0.0464 (17)
P1	0.0377 (5)	0.0521 (5)	0.0318 (4)	−0.0119 (4)	0.0073 (3)	−0.0019 (4)

### Geometric parameters (Å, °)

**Table:**

B1—O3	1.339 (5)	C14—H14	0.93
B1—O2	1.359 (4)	C15—C16	1.362 (7)
B1—C19	1.574 (5)	C15—H15	0.93
C1—C6	1.374 (5)	C16—C17	1.368 (7)
C1—C2	1.379 (5)	C16—H16	0.93
C1—P1	1.798 (3)	C17—C18	1.381 (6)
C2—C3	1.377 (5)	C17—H17	0.93
C2—H2	0.93	C18—H18	0.93
C3—C4	1.362 (6)	C19—C24	1.388 (5)
C3—H3	0.93	C19—C20	1.391 (5)
C4—C5	1.359 (6)	C20—C21	1.393 (6)
C4—H4	0.93	C20—C25	1.494 (6)
C5—C6	1.375 (5)	C21—C22	1.368 (7)
C5—H5	0.93	C21—H21	0.93
C6—H6	0.93	C22—C23	1.375 (7)
C7—C8	1.360 (5)	C22—C26	1.531 (6)
C7—C12	1.374 (5)	C23—C24	1.383 (6)
C7—P1	1.794 (3)	C23—H23	0.93
C8—C9	1.370 (7)	C24—C27	1.505 (6)
C8—H8	0.93	C25—H25A	0.96
C9—C10	1.339 (7)	C25—H25B	0.96
C9—H9	0.93	C25—H25C	0.96
C10—C11	1.356 (7)	C26—H26A	0.96
C10—H10	0.93	C26—H26B	0.96
C11—C12	1.366 (6)	C26—H26C	0.96
C11—H11	0.93	C27—H27A	0.96
C12—H12	0.93	C27—H27B	0.96
C13—C14	1.377 (5)	C27—H27C	0.96
C13—C18	1.383 (5)	O1—P1	1.479 (2)
C13—P1	1.801 (3)	O2—H2A	0.82
C14—C15	1.376 (6)	O3—H3A	0.82
			
O3—B1—O2	118.7 (3)	C16—C17—C18	119.7 (4)
O3—B1—C19	119.0 (3)	C16—C17—H17	120.1
O2—B1—C19	122.3 (3)	C18—C17—H17	120.1
C6—C1—C2	118.8 (3)	C17—C18—C13	120.5 (4)
C6—C1—P1	117.9 (3)	C17—C18—H18	119.8
C2—C1—P1	123.3 (3)	C13—C18—H18	119.8
C3—C2—C1	120.2 (4)	C24—C19—C20	118.8 (3)
C3—C2—H2	119.9	C24—C19—B1	120.6 (3)
C1—C2—H2	119.9	C20—C19—B1	120.5 (3)
C4—C3—C2	120.5 (4)	C19—C20—C21	119.7 (4)
C4—C3—H3	119.8	C19—C20—C25	121.1 (4)
C2—C3—H3	119.8	C21—C20—C25	119.2 (4)
C5—C4—C3	119.6 (4)	C22—C21—C20	121.6 (5)
C5—C4—H4	120.2	C22—C21—H21	119.2
C3—C4—H4	120.2	C20—C21—H21	119.2
C4—C5—C6	120.7 (4)	C21—C22—C23	118.2 (4)
C4—C5—H5	119.6	C21—C22—C26	120.7 (6)
C6—C5—H5	119.6	C23—C22—C26	121.1 (6)
C1—C6—C5	120.2 (4)	C22—C23—C24	121.8 (5)
C1—C6—H6	119.9	C22—C23—H23	119.1
C5—C6—H6	119.9	C24—C23—H23	119.1
C8—C7—C12	117.7 (4)	C23—C24—C19	119.9 (4)
C8—C7—P1	124.3 (3)	C23—C24—C27	119.6 (4)
C12—C7—P1	117.9 (3)	C19—C24—C27	120.6 (4)
C7—C8—C9	120.5 (4)	C20—C25—H25A	109.5
C7—C8—H8	119.8	C20—C25—H25B	109.5
C9—C8—H8	119.8	H25A—C25—H25B	109.5
C10—C9—C8	121.6 (5)	C20—C25—H25C	109.5
C10—C9—H9	119.2	H25A—C25—H25C	109.5
C8—C9—H9	119.2	H25B—C25—H25C	109.5
C9—C10—C11	118.7 (5)	C22—C26—H26A	109.5
C9—C10—H10	120.7	C22—C26—H26B	109.5
C11—C10—H10	120.7	H26A—C26—H26B	109.5
C10—C11—C12	120.7 (5)	C22—C26—H26C	109.5
C10—C11—H11	119.7	H26A—C26—H26C	109.5
C12—C11—H11	119.7	H26B—C26—H26C	109.5
C11—C12—C7	120.8 (4)	C24—C27—H27A	109.5
C11—C12—H12	119.6	C24—C27—H27B	109.5
C7—C12—H12	119.6	H27A—C27—H27B	109.5
C14—C13—C18	118.6 (3)	C24—C27—H27C	109.5
C14—C13—P1	123.4 (3)	H27A—C27—H27C	109.5
C18—C13—P1	118.1 (3)	H27B—C27—H27C	109.5
C15—C14—C13	120.8 (4)	B1—O2—H2A	109.5
C15—C14—H14	119.6	B1—O3—H3A	109.5
C13—C14—H14	119.6	O1—P1—C7	112.18 (15)
C16—C15—C14	120.0 (4)	O1—P1—C1	111.68 (14)
C16—C15—H15	120	C7—P1—C1	107.33 (15)
C14—C15—H15	120	O1—P1—C13	111.86 (15)
C15—C16—C17	120.4 (4)	C7—P1—C13	105.64 (15)
C15—C16—H16	119.8	C1—P1—C13	107.82 (15)
C17—C16—H16	119.8		
			
C6—C1—C2—C3	−0.6 (7)	B1—C19—C20—C25	4.8 (6)
P1—C1—C2—C3	178.9 (4)	C19—C20—C21—C22	1.2 (6)
C1—C2—C3—C4	1.6 (8)	C25—C20—C21—C22	−179.8 (4)
C2—C3—C4—C5	−1.9 (8)	C20—C21—C22—C23	−2.8 (7)
C3—C4—C5—C6	1.2 (7)	C20—C21—C22—C26	176.5 (4)
C2—C1—C6—C5	−0.1 (6)	C21—C22—C23—C24	2.5 (7)
P1—C1—C6—C5	−179.6 (3)	C26—C22—C23—C24	−176.7 (4)
C4—C5—C6—C1	−0.2 (6)	C22—C23—C24—C19	−0.7 (6)
C12—C7—C8—C9	−1.2 (7)	C22—C23—C24—C27	179.9 (4)
P1—C7—C8—C9	−177.9 (4)	C20—C19—C24—C23	−1.0 (5)
C7—C8—C9—C10	−0.8 (10)	B1—C19—C24—C23	175.9 (3)
C8—C9—C10—C11	1.5 (10)	C20—C19—C24—C27	178.4 (4)
C9—C10—C11—C12	−0.2 (9)	B1—C19—C24—C27	−4.7 (5)
C10—C11—C12—C7	−1.8 (8)	C8—C7—P1—O1	132.5 (4)
C8—C7—C12—C11	2.5 (7)	C12—C7—P1—O1	−44.2 (3)
P1—C7—C12—C11	179.4 (4)	C8—C7—P1—C1	9.4 (4)
C18—C13—C14—C15	0.4 (5)	C12—C7—P1—C1	−167.3 (3)
P1—C13—C14—C15	−178.1 (3)	C8—C7—P1—C13	−105.4 (4)
C13—C14—C15—C16	−0.6 (6)	C12—C7—P1—C13	77.9 (3)
C14—C15—C16—C17	−0.2 (7)	C6—C1—P1—O1	−27.1 (3)
C15—C16—C17—C18	1.1 (7)	C2—C1—P1—O1	153.4 (3)
C16—C17—C18—C13	−1.3 (7)	C6—C1—P1—C7	96.2 (3)
C14—C13—C18—C17	0.5 (6)	C2—C1—P1—C7	−83.3 (4)
P1—C13—C18—C17	179.1 (3)	C6—C1—P1—C13	−150.4 (3)
O3—B1—C19—C24	−103.4 (5)	C2—C1—P1—C13	30.1 (4)
O2—B1—C19—C24	76.2 (5)	C14—C13—P1—O1	145.3 (3)
O3—B1—C19—C20	73.4 (5)	C18—C13—P1—O1	−33.3 (3)
O2—B1—C19—C20	−106.9 (4)	C14—C13—P1—C7	23.0 (3)
C24—C19—C20—C21	0.7 (5)	C18—C13—P1—C7	−155.6 (3)
B1—C19—C20—C21	−176.2 (3)	C14—C13—P1—C1	−91.6 (3)
C24—C19—C20—C25	−178.3 (4)	C18—C13—P1—C1	89.9 (3)

### Hydrogen-bond geometry (Å, °)

**Table:**

Cg4 is the centroid of the C19–C24 benzene ring.

**Table:**

*D*—H···*A*	*D*—H	H···*A*	*D*···*A*	*D*—H···*A*
O2—H2A···O1	0.82	1.84	2.645 (3)	168
O3—H3A···O2^i^	0.82	1.99	2.795 (4)	169
C4—H4···O1^ii^	0.93	2.41	3.326 (4)	167
C6—H6···Cg4	0.93	2.88	3.728 (4)	152
C15—H15···Cg4^iii^	0.93	2.69	3.602 (5)	168

Symmetry codes: (i) −*x*+1, −*y*, −*z*; (ii) *x*, −*y*+1/2, *z*+1/2; (iii) *x*+1, *y*, *z*.
